# Valorization of Soybean Peel-Derived Humins for Carbon Dot (CD) Production

**DOI:** 10.3390/ma18081865

**Published:** 2025-04-18

**Authors:** Onofrio Losito, Thomas Netti, Veronika Kost, Cosimo Annese, Lucia Catucci, Tatiana Da Ros, Vincenzo De Leo, Lucia D’Accolti

**Affiliations:** 1 Department of Chemistry, University of Bari Aldo Moro, Via Orabona 4, 70126 Bari, Italy; onofrio.losito@uniba.it (O.L.); th.netti97@gmail.com (T.N.); lucia.catucci@uniba.it (L.C.); lucia.daccolti@uniba.it (L.D.); 2 Department of Chemical and Pharmaceutical Sciences, University of Trieste, Via Giorgieri 1, 34127 Trieste, Italy; veronika.kost@units.it (V.K.); daros@units.it (T.D.R.); 3 Department of Life Science, Health and Health Professions, Link Campus University, Via del Casale di S. Pio V 44, 00165 Roma, Italy; c.annese@unilink.it; 4 CNR-ICCOM S.S. Bari, c/o Department of Chemistry, University of Bari Aldo Moro, Via Orabona 4, 70126 Bari, Italy; 5 CNR-IPCF S.S. Bari, c/o Department of Chemistry, University of Bari Aldo Moro, Via Orabona 4, 70126 Bari, Italy

**Keywords:** carbon dots, humins, biomass, soybean peels

## Abstract

Over the past few decades, awareness has risen substantially about the limitations of non-renewable resources and the environmental challenges facing the chemical industry. This has necessitated a transition toward renewable resources, such as lignocellulosic biomass, which is among the most abundant renewable carbon sources on the planet. Lignocellulosic biomass represents a significant yet often underutilized source of fermentable sugars and lignin, with potential applications across multiple sectors of the chemical industry. The formation of humins (polymeric byproducts with a complex conjugated network, comprising furanic rings and various functional groups, including ketones) occurs inevitably during the hydrothermal processing of lignocellulosic biomass. This study presents the use of humin byproducts derived from soybean peels for the production of fluorescent carbon dots (CDs). A comparison between sonochemical and thermochemical methods was conducted for the synthesis of this nanomaterial. The obtained nanoparticles were characterized in terms of size, morphology (TEM, DLS), and Z-potential. Subsequently, the spectroscopic properties of the prepared CDs were studied using absorption and emission spectroscopy. In particular, the CDs displayed a blue/cyan fluorescence under UV irradiation. The emission properties were found to be dependent on the excitation wavelength, shifting to longer wavelengths as the excitation wavelength increased. The carbon dots that exhibited the most favorable photochemical properties (QY = 2.5%) were those produced through a sonochemical method applied to humins obtained from the dehydration of soybean husks with phosphoric acid and prior treatment.

## 1. Introduction

In recent decades, awareness of the limitations of natural resources and environmental constraints surrounding the chemical industry has grown significantly. The linear production model, characterized by the consumption of precious resources followed by the production of waste, has been identified as the main factor that significantly contributes to climate change, biodiversity loss, and the shortage of food, water, and energy resources.

**Figure 1:**
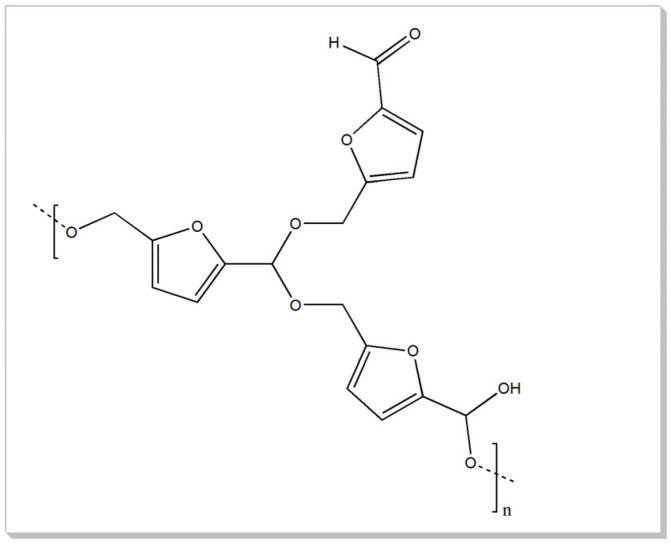
General structure of humins.

A shift toward viewing waste as a resource is essential for fostering a circular economy. This eco-design consideration enhances the potential for the full recycling of both feedstocks and products [1]. Lignocellulosic biomass, one of Earth’s most abundant sources of renewable carbon, represents a significant and often underutilized source of fermentable sugars with a global annual production estimated at 2 × 10^11^ tons [2]. This biomass derives from various waste streams across industries, forestry, agriculture, and municipalities. and is primarily composed of cellulose, hemicellulose, and lignin, a polymeric phenolic compound [3,4,5]. The carbohydrate components within this biomass can be converted via acid-catalyzed dehydration into valuable chemical intermediates, including furfural, 5-hydroxymethylfurfural (HMF), and levulinic acid (LA), which are pivotal to industries such as fuel, polymer, and solvent production [4,5,6]. Numerous factors, including a biomass’s physicochemical, structural, and compositional properties, hinder a biomass’s conversion to fermentable sugars and other top-value chemicals. Pretreatment processes can mitigate some of these barriers by partially removing lignin and hemicellulose, reducing cellulose crystallinity, and increasing biomass porosity. Pretreatment processes are vital in overcoming these barriers by facilitating the removal of lignin and hemicellulose, reducing the crystallinity of cellulose, and enhancing the porosity of biomass. Among the various pretreatment methods, chemical pretreatment using inorganic acids such as hydrochloric acid (HCl) and sulfuric acid (H_2_SO_4_) has gained attention for its effectiveness in improving cellulose biodegradability. This is achieved through the selective removal of lignin and hemicellulose, as well as reductions in the degree of polymerization and crystallinity of the cellulose fraction [4,7]. A major challenge in acid-catalyzed biomass conversion is the formation of humins, which are polymeric byproducts with a complex, conjugated network structure that includes furanic rings and various functional groups such as ketones, aldehydes, and aromatic alcohols (Figure 1). Numerous studies have sought to unravel the mechanisms behind humin formation, examining diverse pathways that lead to distinct products [8,9,10,11,12,13].

**Figure 2:**
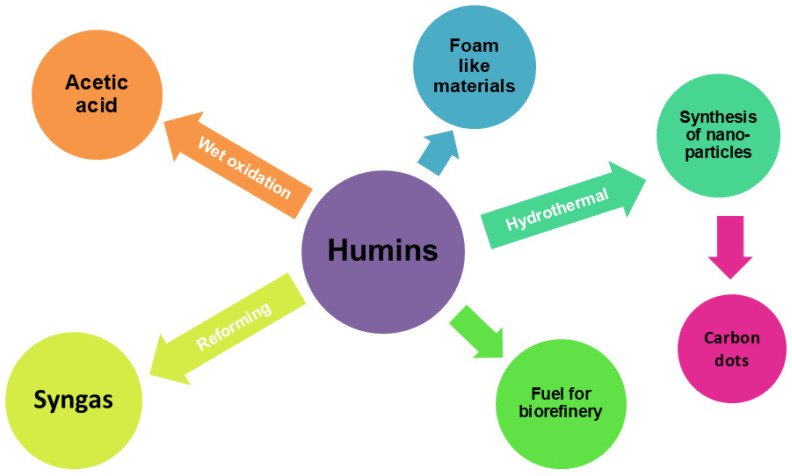
Main applications of humins [13,14,16,19].

Humin formation is inevitable due to the thermodynamic characteristics of the acid-catalyzed reaction, and its extent depends on the initial feedstock concentration; specifically, a higher initial feedstock concentration leads to higher humin yields. The humin yield is usually 20–50%, with a carbon content of 55–65% [12]. The latter means that much of the carbon of the feedstock would be lost if not used to obtain any interesting product. As they are a particularly abundant by-product, exploring new ways to utilize humins to prepare new high-added-value products is essential. Currently, humins are mainly used as fuel for heat generation owing to their carbon rich characteristics, but they have the potentiality to be converted into oils and syngas [13] through thermochemical processes, into acetic acid [14] by means of wet oxidation, or into valuable composites/materials [15]. Recent applications of humins in developing new materials have included a synthesis of a macroporous foam-like substance [16], along with a few approaches to nanomaterials [17,18] (Figure 2).

This work focuses on a poorly considered application of humins and their valorization for preparing carbon-based nanomaterials with high added value, in particular carbon dots (CDs). Two types of processes were compared for dot synthesis starting from humins from soybean peels: (1) hydrothermal and (2) heat treatment/sonication.

CDs are generally less than 10 nm in size, and compared to the classic, inorganic-based quantum dots, they possess some interesting properties such as good photostability, good biocompatibility, low cytotoxicity, easy surface modification, and high chemical inertness. The structure of CDs is composed of carbon- and oxygen–nitrogen-based sp^2^/sp^3^ groups or polymeric aggregations. “CDs” is in fact a fairly general definition that includes graphene quantum dots, carbon nanodots, and polymer dots. Graphene quantum dots have one or a few layers of graphene and chemical groups connected on the edges and therefore have an anisotropic structure with lateral dimensions larger than their height. Carbon nanodots instead have a spherical structure and are divided into carbon nanoparticles (without a crystal lattice) and carbon quantum dots (with a crystal lattice). Finally, polymer dots are aggregated or cross-linked polymers generally prepared from polymers or monomers [20]. The unique fluorescence characteristic of nearly all CDs is that their emission spectrum depends on the excitation wavelength, shifting to longer wavelengths (bathochromic shift) as the excitation wavelength increases. Thanks to these properties, CDs have found their way into several application fields: cellular imaging, in vivo imaging, drug delivery, fluorescence sensing, photocatalysis, the production of multicolor light-emitting diodes (LEDs), and energy conversion and storage [19].

Compared with many other methods, sonochemical preparation is an environmentally friendly method with advantages such as easy and mild experimental conditions, and it also allows the formulation of CDs and doped CDs with controlled physicochemical properties and lower toxicity [21]. Different substrates have been used (such as carbon soot [22], graphite [23], and carbohydrates [24]) to obtain water- and oil-soluble dots with quantum yields ranging from 1.6 to 30% [22,23,24].

The findings of this study reveal a groundbreaking advancement in the existing literature, as they demonstrate the innovative use of humins derived from soybean peels in the synthesis of CSs. Soybeans are one of the most important crops worldwide, as they are used for human consumption, animal feed production, lecithin production, biofuels, and more. The worldwide production of soybeans was around 395 million tons in 2024 according to FAO, and is a constantly increasing market, as the foreseen production for 2025 is 421 million tons, as the fourth commodity in the agriculture market [25]. All of this generates a large amount of waste material, the valorization of which is essential to mitigate, at least in part, the significant environmental impact of this crop. In addition, some of the authors have recently reported that soybean peels have proven to be the most reactive biomass for the synthesis of high-value chemicals [4]. By harnessing the unique properties of humins extracted from soybean peels, this research opens up new avenues for the sustainable and eco-friendly production of carbon-based nanomaterials, highlighting the versatility of agricultural waste and its valuable contributions to nanotechnology. The implications of these findings could pave the way for further exploration into the use of other biomass sources, ultimately promoting a circular economy in the field of materials science.

## 2. Materials and Methods

### 2.1. Materials

Ethyl acetate (>99%) was purchased by Honeywell, and phosphoric acid (85%) and acetic acid (glacial) were purchased from Sigma-Aldrich (St. Louis, MO, USA). All the reagents and solvents were used as received, without any further treatment.

### 2.2. Methods

#### 2.2.1. Humin Production from Soybean Peels

Three distinct samples of humins were synthesized from soybean peels through varying pretreatment and hydrothermal conditions. Initially, 250 mg aliquots of soybean peels were prepared, with one aliquot suspended in a 4 M acetic acid aqueous solution and then heated in a stainless-steel autoclave at 200 °C for 2 h (H1). The second aliquot of 250 mg of soybean peels was suspended in 100 mL of a 15% *w*/*w* aqueous solution of phosphoric acid (H_3_PO_4_) and directly heated in the autoclave at 200 °C for 2 h (H2). The third aliquot, similarly to the previous one, was suspended in 100 mL of a 15% *w*/*w* aqueous H_3_PO_4_ solution, but after having been subjected to a preliminary heating step at 80 °C for 2 h. Following this pretreatment, the mixture was transferred to a stainless-steel autoclave and subjected to hydrothermal treatment at 200 °C for an additional 2 h (H3). In all cases, following the hydrothermal processing, the mixtures were allowed to cool, after which they were subjected to filtration and/or centrifugation to isolate the solid humins. The resulting humins were subsequently dried and weighed, yielding a mass recovery ranging from 20% to 80%.

#### 2.2.2. Synthesis of CDs by Hydrothermal Method

**Figure 3:**
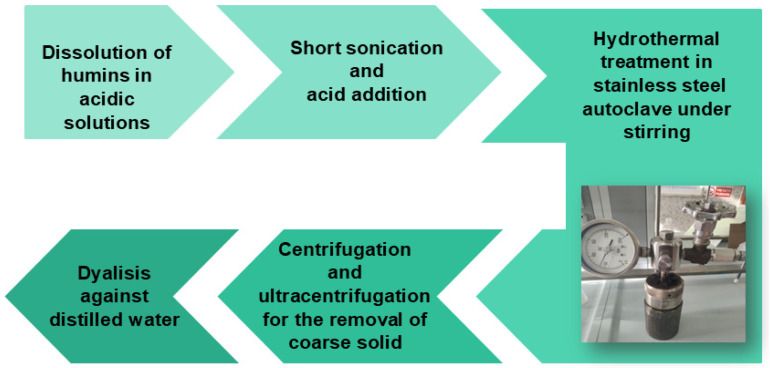
Schematic illustration of hydrothermal process.

The hydrothermal method for synthesizing carbon dots (CDs) utilizes an acidic solution heated to high temperatures to promote the carbonization of organic precursors in an aqueous medium (Figure 3). In this process, carbon-rich materials (such as sugars, biomass, or other organic compounds) are dissolved in water and subjected to elevated temperatures and pressures within a sealed autoclave. This environment facilitates the formation of nanoscale carbon structures that exhibit quantum confinement effects, resulting in distinct optical properties, including photoluminescence. The hydrothermal method is particularly favored for its straightforward approach, eco-friendliness, and capacity to generate CDs with adjustable sizes and surface functionalities. In all tests, phosphoric acid was selected for CD synthesis.

##### Hydrothermal Synthesis of CDs from Humins H1

A sample of 25 mg of humins (obtained from the reaction of soybean peels with acetic acid, H1) was dispersed in 20 mL of distilled water and the mixture was sonicated for 10 min. Then, 0.1 g of phosphoric acid was added and the mixture was heated under stirring at 200 °C in an autoclave for 3 h. After the reaction was completed, the autoclave was cooled, and the mixture was subjected to centrifugation at 4000 rpm for 30 min to remove the coarse solid residue, obtaining a yellow supernatant solution. The solution was subjected to ultracentrifugation at 20,000 rpm for 3 h and the supernatant was recovered (this test was named T1A). The same procedure was applied to another 20 mg of humins with a reaction time of 6 h at 200 °C (this test was named T1B).

##### Hydrothermal Synthesis of CDs from Humins H2

First, 25 mg of humins (obtained from the dehydration reaction of soybean peels with phosphoric acid, H2) were dispersed in 10 mL of distilled water and the mixture was sonicated for 10 min. Then, 0.1 g of phosphoric acid was added and the mixture was heated under agitation at 200 °C in an autoclave for 3 h. After the reaction was completed, the autoclave was cooled and the mixture was subjected to centrifugation at 4000 rpm for 30 min to remove the coarse solid residue, obtaining a yellow supernatant solution. The solution was subjected to ultracentrifugation at 20,000 rpm for 3 h and the supernatant was recovered; this test was named T2A. The same procedure was applied to another 25 mg of humins with a reaction time at 200 °C of 6 h (T2B).

##### Hydrothermal Synthesis of CDs from Humins H3

First, 25 mg of humins (obtained from the dehydration reaction of soybean peels with phosphoric acid and pretreatment, H3) were dispersed in 10 mL of distilled water and the mixture was sonicated for 10 min. Then, 0.1 g of phosphoric acid was added and the mixture was heated under agitation at 200 °C in an autoclave for 3 h. After the reaction was completed, the autoclave was cooled and the mixture was subjected to centrifugation at 4000 rpm for 30 min to remove the coarse solid residue, obtaining a yellow supernatant solution. The solution was subjected to ultracentrifugation at 20,000 rpm for 3 h and the supernatant was recovered; this test was named T3A. The same procedure was applied to another 25 mg of humins with a reaction time at 200 °C of 6 h (T3B).

#### 2.2.3. Synthesis of CDs by Low-Temperature Carbonization and Ultrasonication

**Figure 4:**
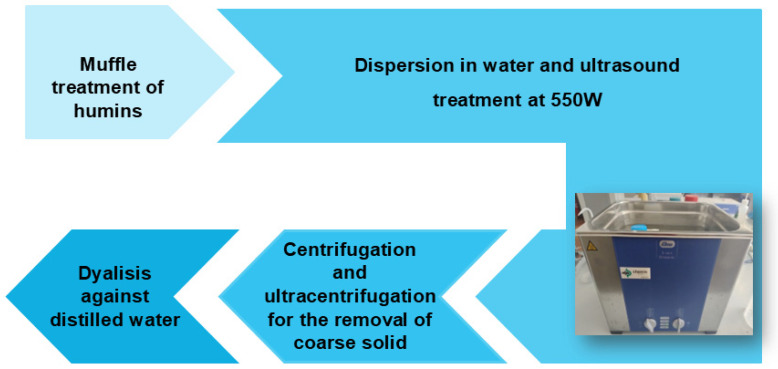
Schematic illustration of sonochemical process.

The sonochemical method for producing carbon quantum dots (CDs) involves using ultrasonic waves to induce chemical reactions in precursor materials, typically carbon-rich organic compounds, or biomass (Figure 4). During sonication, cavitation bubbles form and collapse, generating localized high temperatures and pressures that lead to the fragmentation of the carbon sources and the formation of nanostructures. This technique is advantageous due to its simplicity, rapid processing time, and ability to produce CDs with tunable sizes and surface functional groups. Additionally, the sonochemical approach is often environmentally friendly, as it can utilize renewable resources and reduces the need for hazardous chemicals, making it possible to use water as a dispersing agent.

##### Sonochemical Synthesis of CDs from Humins H1

Humins (obtained from dehydration of soybean peels and acetic acid, H1) were muffle-treated at 250 °C for 2 h (in air atmosphere). A sample of 50 mg of the residue obtained was dispersed in 5.5 mL of distilled water and was subjected to sonication at 550 W (ultrasonic bath) for 30 min; the black mixture obtained after sonication was centrifuged at 4000 rpm to remove coarse residues and the supernatant was subjected to filtration (with 0.2 μm filter). The resulting solution was ultracentrifuged for 30 min at 20,000 rpm. This test was named T4.

##### Sonochemical Synthesis of CDs from Humins H2

Humins (obtained from dehydration of soybean peels and phosphoric acid, H2) were muffle-treated at 250 °C for 2 h (in air atmosphere). A sample of 50 mg of the residue obtained was dispersed in 5.5 mL of distilled water and was subjected to sonication at 550 W (ultrasonic bath) for 30 min; the black mixture obtained after sonication was centrifuged at 4000 rpm to remove coarse residues and the supernatant was subjected to filtration (with 0.2 μm filter). The resulting solution was ultracentrifuged for 30 min at 20,000 rpm. This test was named T5.

##### Sonochemical Synthesis of CDs from Humins H3

Humins (obtained from dehydration of soybean peels with phosphoric acid and pretreatment, H3) were muffle-treated at 250 °C for 2 h (in an air atmosphere). A sample of 50 mg of the residue obtained was dispersed in 5.5 mL of distilled water and was subjected to sonication at 550 W (ultrasonic bath) for 30 min; the black mixture obtained after sonication was centrifuged at 4000 rpm to remove coarse residues and the supernatant was subjected to filtration (with 0.2 μm filter). The resulting solution was ultracentrifuged for 30 min at 20,000 rpm and an aliquot of the post ultra-centrifugal supernatant was dialyzed for 3 h against distilled water using a membrane with 3.5 KDa MWCO; this test was named **T6**.

All the materials and the principal features of their preparation are reported in Table 1.

### 2.3. Characterization of Humins and CDs

The particle size of CDs was determined before and after dialysis by dynamic light scattering (DLS) analysis using a Zetasizer Nano ZS (Malvern Panalytical Ltd., Malvern, UK). The Z-potential determination was performed using laser Doppler anemometry (Zetasizer Nano ZS, Malvern Panalytical Ltd., Malvern, UK) after dilution in distilled water (1:10).

CD morphology was investigated by transmission electron microscopy (TEM) (Thermo Fisher Scientific, Waltham, MA, USA). TEM images were collected using an EM 208 microscope (Philips, Eindhoven, The Netherlands) equipped with a Quemesa camera (Olympus Soft Imaging Solutions, Tokyo, Japan). The images were acquired at a voltage of 100 kV using RADIUS software version 2.1. Size statistical analysis (CD average size and size distribution) was performed using a freeware Image J analysis program version 1.53e, java 1.8.0_172, 64-bit (National Institutes of Health, Bethesda, MD, USA).

UV–Vis absorption spectra of CD suspensions were acquired by a Cary 5000 (Agilent Technologies, Santa Clara, CA, USA) ultraviolet–visible, double-beam spectrophotometer in the range of 200–600 nm by applying the baseline correction, while fluorescence spectra were recorded by a Varian Cary Eclipse Spectrofluorometer (Agilent Technologies, Santa Clara, CA, USA).

Attenuated total reflectance Fourier transform infrared (ATR-FTIR) spectroscopy measurements of humins and CDs were acquired using a Perkin Elmer UATR-Two spectrophotometer instrument (PerkinElmer, Waltham, MA, USA) equipped with a single reflection diamond ATR crystal (refractive index of 2.4). Spectra were acquired with 32 scans in the range of 4000–600 cm^−1^ by applying both the baseline and the ATR corrections.

Thermogravimetric (TGA) analyses of humins were carried out with a Perkin Elmer Pyris 1 TGA under nitrogen flow.

## 3. Results and Discussion

### 3.1. Humin Characterization

#### 3.1.1. FTIR-ATR Analyses

**Figure 5:**
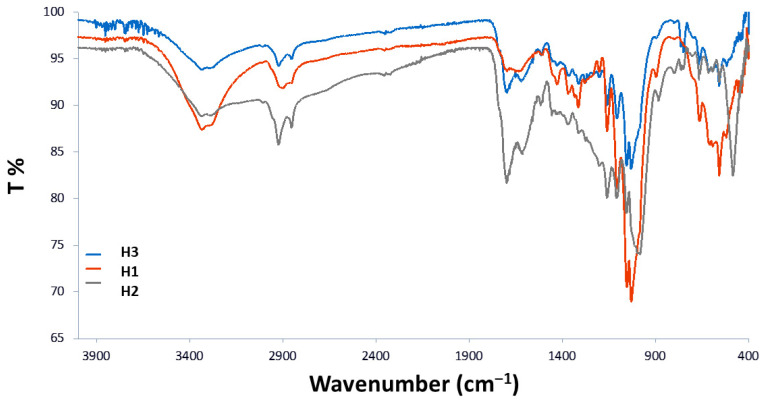
ATR-FTIR overlapped spectra of humins H1–H3 obtained from soybean peels with H_3_PO_4_ and pretreatment, acetic acid and H_3_PO_4_.

The chemical characterization of the humins used in this work for the synthesis of CDs was carried out using FTIR-ATR analysis to identify their structural features. As shown in Figure 5, the FTIR profiles of humin samples were similar. The broad band at the 3040–3679 cm^−1^ range was assigned to -OH stretching vibrations, while the split peak at 2852/2924 cm^−1^ indicated the -CH_2_- stretch. The strong signal at 1699 cm^−1^ was associated with the carbonyl -C=O stretching vibration, characteristic of α,β-saturated aldehydes [26]. The C=C stretch of the furanic ring appeared at 1622 cm^−1^, while the signal detected at 1426 cm^−1^ was assigned to the -C-OH in-plane bending. Further, multiple signals observed at 1323 cm^−1^, 1315 cm^−1^, and 1159 cm^−1^ corresponded to C-O and C-O-C (furan) bonds, respectively [26,27]. The following signals in the fingerprint area of 1030–1054 cm^−1^ were associated with hydroxyl group -OH bending, while the out-of-plane bending of C-H furanic rings was detected in the 750–800 cm^−1^ range [26,27]. Table 2 summarizes the main signals recorded in the three spectra.

#### 3.1.2. TGA Analyses

**Figure 6:**
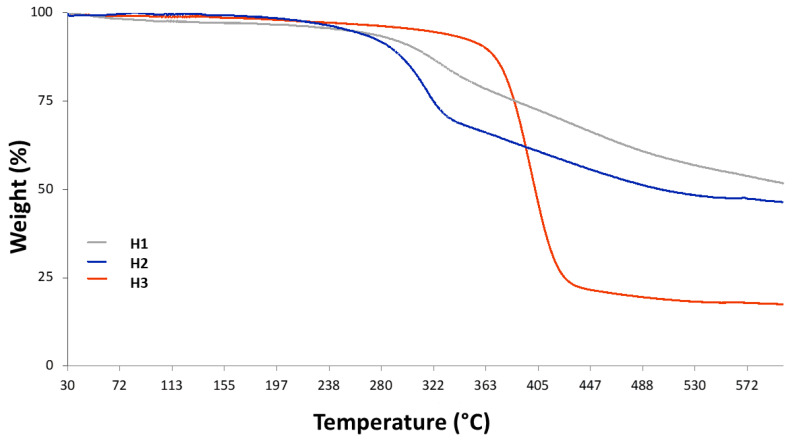
TGA analyses of humins H1–H3 obtained from soybean peels.

TGA analysis of humins has been carried out from 30 °C to 600 °C using a temperature ramp of 10 °C/min. The profiles obtained from the TGA analyses of the various humic samples (Figure 6) show that they all exhibit an inflection between 300 °C and 400 °C. This is a classical behavior of organic carbonaceous materials. However, it is possible to appreciate a relevant difference between the humins deriving from the simple acidic and thermal treatment (H1 and H2) and the one pretreated with phosphoric acid at 80 °C for 2 h (H3). While the stability of the first two is quite low at already 250–300 °C, as if they start losing functional groups on their edges, the third sample, having been subjected to a harsher process, results in being more stable at this temperature and its weight loss profile shows a defined variation at 400 °C, with a rather stable residual between 450 °C and 600 °C (21 and 17.7%, respectively). The behavior of the other two samples at the same temperatures is quite different (66.3 and 51.8 for H1 and 55.8 and 46.4% for H2), demonstrating that the samples are less thermosensitive but, at the same time, that they did not reach stability at 600 °C. Thus, it is noticeable that the longer exposure of soybeans peels to H_3_PO_4_ reduced the thermal stability of humins (H3) at high temperatures, as confirmed by TGA results. In particular, the drastic weight loss indicated the presence of more oxygen-containing functionalities in the humin material after additional acidic treatment in comparison with H1 and H2, even though the more instable carboxylic groups on the surface are more abundant on H1 and H2 as demonstrated by the weight loss of those preparations at low temperatures (250–350 °C).

### 3.2. Synthesis of CDs by Hydrothermal Method

**Figure 7:**
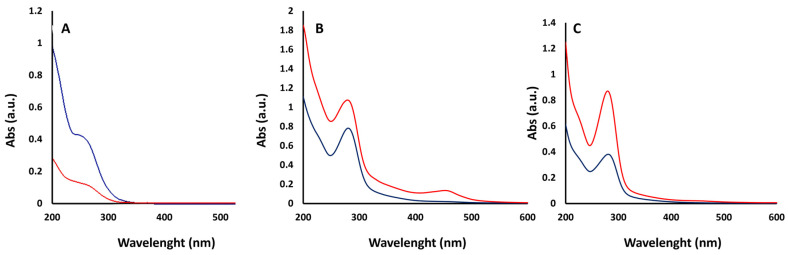
UV-vis absorption spectra of (**A**) T1A (blue) and T1B (red); (**B**) T2A (blue) and T2B (red); (**C**) T3A (blue) and T3B (red). All samples are measured after ultracentrifugation.

The hydrothermal synthesis of CDs was carried out at 200 °C, since preliminary experiments indicated that at lower temperatures no fluorescence was observed in the solutions, suggesting that the formation of CDs did not occur in these conditions, while at higher temperatures no significant increase in fluorescence was observed. This result is similar to that reported by Zhang et al. [17], who even found a decrease in fluorescence at temperatures above 200 °C. The effect of reaction time was evaluated by carrying out the synthesis at 3 h and 6 h as representative of a “short” and a “long” reaction time. Therefore, humins obtained from different processes (acetic acid T1; H_3_PO_4_-T2, and H_3_PO_4_ with pretreatment T3) have been briefly sonicated and treated in a stainless-steel autoclave for 3 h (A tests) and 6 h (B tests) in an acidic environment using phosphoric acid. Phosphoric acid was selected for hydrothermal synthesis because of its higher acidity compared to other acids, which is capable of effectively promoting the carbonization of humin precursors. The samples were then ultracentrifuged, and UV spectra of the supernatant were registered (Figure 7). CDs generally exhibit a well-defined optical absorption in the UV range, with a tail into the visible region of the spectrum. In accordance with this behavior, all samples in Figure 7 showed this type of absorption spectrum, with a strong absorption peak around 280 nm, typically attributed to a π-π* transition of the aromatic C–C bonds.

The fluorescence of these samples appeared to be weak in intensity, preventing the calculation of quantum yield (Table 3). Thus, no further investigation was carried out on these CDs.

### 3.3. Synthesis of CDs by Ultrasonic Treatment

**Figure 8:**
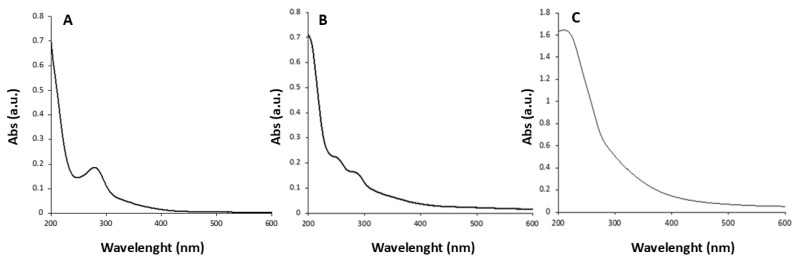
UV-vis absorption spectra of T4 (**A**), T5 (**B**), and T6 (**C**) after ultracentrifugation.

This synthetic approach involved treating the humin samples in a muffle furnace at 250 °C for 2 h (in an air atmosphere). Subsequently, an aliquot of each obtained sample residue was dispersed in distilled water and sonicated at 550 W in an ultrasonic bath for 30 min at room temperature. The black mixture obtained after sonication was centrifuged at 4000 rpm to remove coarse residues and the supernatant was filtered. The resulting solution was ultracentrifuged for 30 min at 20,000 rpm, and finally the supernatant was analyzed by UV-vis spectroscopy (Figure 8).

After ultracentrifugation, the T4 and T5 samples showed very low fluorescence; thus, no further investigation has been carried out. Instead, T6 showed appreciable fluorescence. The improvement in photoluminescent properties of T6 compared to T4 and T5 might be associated with the lower thermal stability of the humic precursor (H3), confirmed by TGA analysis.

At the same time, the superior PL characteristics of T6 towards other carbon dot series (T1–T3) indicated the efficiency of the sonication-assisted approach over hydrothermal conditions.

To discharge the possibility that the positive luminescence response was due to the presence of molecular species, a process of purification was performed. An aliquot of the T6 sample was subjected to dialysis against distilled water using a membrane with MWCO 3.5 KDa for 3 h.

**Figure 9:**
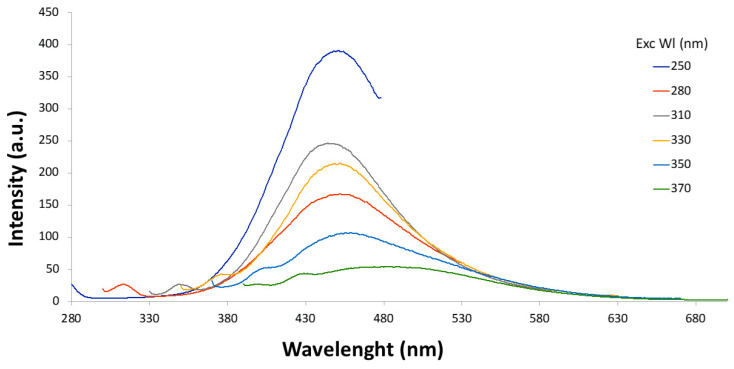
Fluorescence spectra recorded progressively increasing excitation wavelengths (from 250 to 370 nm) as prepared T6 sample.

**Figure 10:**
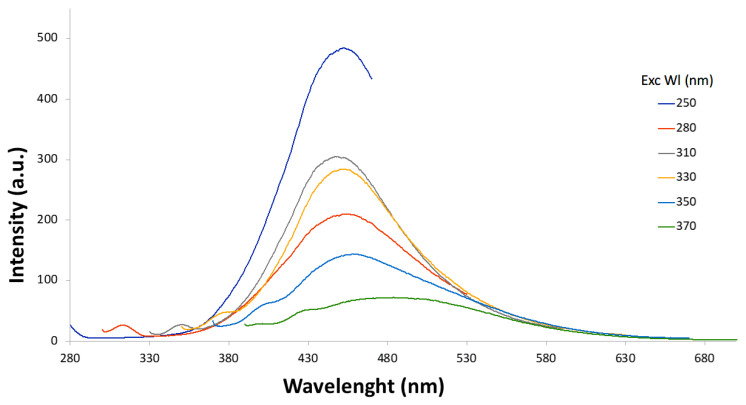
Fluorescence spectra recorded progressively increasing excitation wavelengths (from 250 to 370 nm) of T6 sample after dialysis purification.

After a short sonication, to prevent aggregation, fluorescence spectra of T6 before and after dialysis were acquired at different wavelengths (Figure 9 and Figure 10), and quantum yield was determined using quinine sulfate as a standard (Table 4).

**Figure 11:**
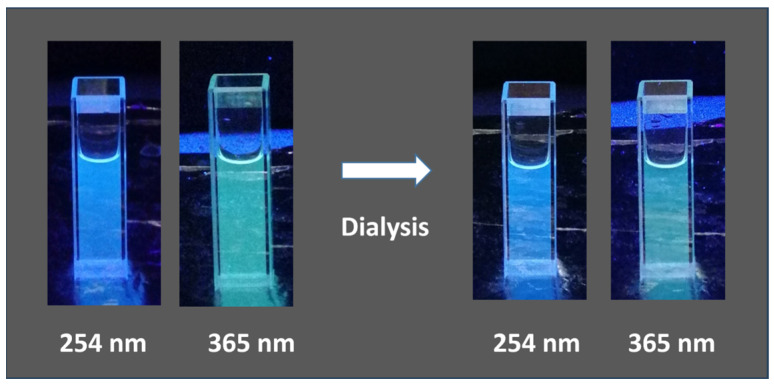
Fluorescence of **T6** sample before and after dialysis purification under UV illumination.

**Figure 12:**
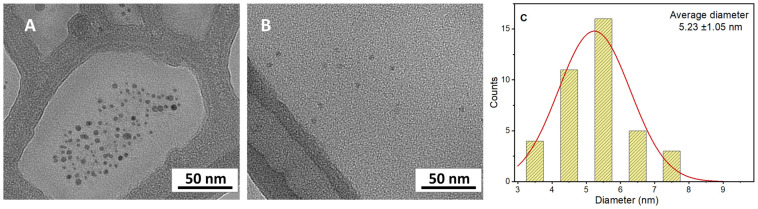
TEM images of as-prepared T6 CDs (**A**), and after dialysis purification (**B**) with corresponding particle size distribution diagram for purified sample (**C**).

The T6 sample displayed a blue/cyan fluorescence under UV irradiation (Figure 11). Excitation at 250 nm resulted in the highest emission intensity. In both Figure 9 and Figure 10, it is possible to notice that the intensity of signal decreases as the excitation wavelength increases and the emission peak undergoes a slight red shift. The dependence of the fluorescence wavelength on the excitation wavelength is a frequently observed phenomenon for CDs, although its interpretation remains debated. Furthermore, it can be observed that the dialysis purification process did not alter the emission properties of the CDs. Quantum yield (QY) for T6 after ultracentrifugation was 2.6%, while after dialysis it was 2.4%. This slight reduction could be justified considering that the dialysis could have removed molecular components contributing to fluorescence. However, this is a top-down approach in preparing the carbon nanomaterials, and the presence of singular fluorescent molecules was minimized with respect to the bottom-up synthetic pathway. The size of “as-prepared” T6 particles was determined by DLS and resulted in 7.3 ± 1.2 nm, while after dialysis, it was 7.8 ± 1.9 nm. Thus, as expected from the spectroscopic behavior, particle size was not affected by the dialysis process. The TEM images highlighted well-dispersed and round-shaped nanostructures (Figure 12A), with sizes in line with those measured by DLS. In addition, TEM images demonstrated that the purification process by dialysis did not cause significant alterations in the CDs nor the aggregation phenomena of the nanoparticles (Figure 12B). Zeta potential measurements returned a value of −42 ± 2 mV for the T6 sample, suggesting good colloidal stability, which is a key factor, for example, in ensuring the uniform distribution of CDs in drug delivery systems.

The scientific literature reports numerous cases of CD synthesis from various biomass sources, although the most numerous examples are related to hydrothermal syntheses. For example, palm shell waste was used to produce CDs with sizes ranging from 4 to 10 nm [28]. Similarly, pomelo peels and bamboo leaves have also been explored for CD production. The majority of CDs derived from bamboo leaves exhibit particle sizes between 3.4 and 4.2 nm, while those from pomelo peels show a size distribution between 2 and 4 nm [29,30]. Recent reviews highlighted the use of several biomass wastes for CD synthesis [31]. Among these, lettuce leaves produced CDs with a QY of 0.38% and an average size of 2.8 nm (Table 5, Entry 1), grape seeds yielded CDs with a QY of 0.275% and an average size of 8.9 nm (Table 5, Entry 2), and sugarcane bagasse resulted in CDs with a QY of 0.092% and a size range from 2 to 8 nm (Table 5, Entry 3) [32,33,34]. Finally, the entries 4 and 5 showed the preparation of CDs from the soybeans and also husk, while, to the best of our knowledge, humins have never been used as precursors before (entries 6 and 7).

These results suggest that the sizes of CDs obtained in this study are consistent with those reported in the literature for biomass-derived CDs. Furthermore, the QYs of the CDs synthesized for the first time from humins—materials that could be classified as real waste—were enhanced through a sonochemical approach. This method resulted in significantly higher QYs than those reported in other studies utilizing waste and biomass as precursor materials.

Generally, QY depends on the synthesis route and the surface chemistry of CDs. As just discussed, as-synthesized CDs derived from biomass sources generally have low QYs, even lower than 1%. Post-synthesis surface modification or passivation is a common and effective way to dramatically increase the QY of CDs [20]. Surface modification has also been used not only to increase the QY but also to tune the PL emission of CDs. For example, the green emission can be changed to a blue emission by surface reduction as previously reported [37]. The CDs obtained here by the sonochemical method have a QY that is already sufficient for cell-labeling experiments but could be appropriately functionalized to adapt them to specific experimental applications.

**Figure 13:**
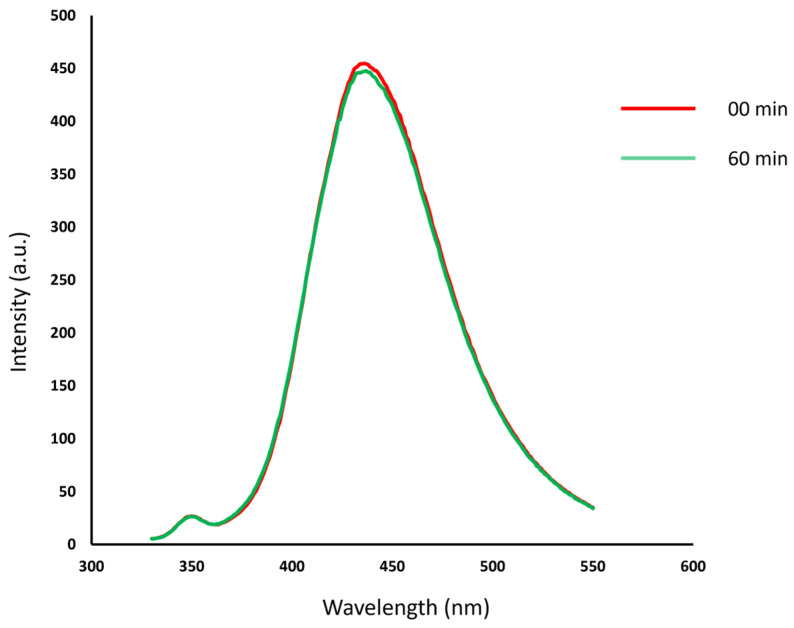
Fluorescence spectra (λ_ex_ = 280 nm) of T6 CDs in aqueous solution at different irradiation times of 365 nm of UV light.

The fluorescence photostability of the CDs is an important parameter for their application in the biological field. The photostability of the T6 CD sample was therefore evaluated using the fluorometric method, as described previously [38]. The CD solution was exposed to 365 nm of UV light for 60 min, after which the fluorescence intensity was measured. The emission peak intensity remained nearly unchanged compared to the original intensity (see Figure 13). This behavior suggests that the CDs synthesized from humins exhibit good photostability, making these nanoparticles promising candidates as fluorescent probes for cell imaging and labeling. These properties may also make them suitable for microscopy, analytical applications, and other uses in the biological field. CDs are particularly suitable for biological applications not only because of their spectroscopic properties but also because toxicity studies conducted previously by various research groups have shown that they have low toxicity in vitro and in vivo. In fact, compared with metal-based quantum dots, CDs are composed of carbon, which is an intrinsically non-toxic element [20].

**Figure 14:**
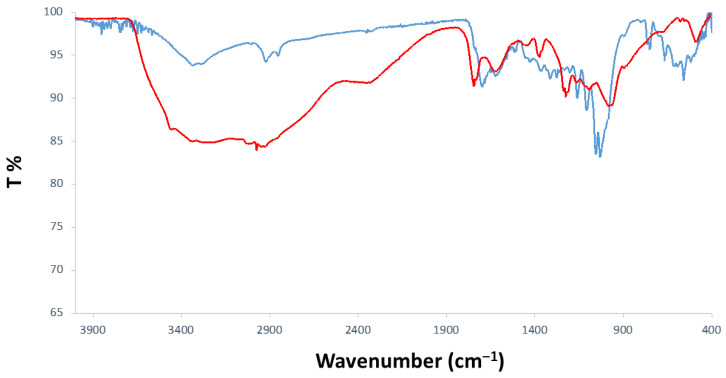
ATR-FTIR overlapped spectra of T6 CDs (red line) and their precursor humins H3 (blue line).

The FTIR spectra of T6 (Figure 14) evidence the successful transformation of the molecular precursor (humins H3) to carbonaceous cores covered with multiple functional groups. Thus, the -OH stretching vibrations appeared in the T6 IR profile as a much broader peak covering the region of 2000–3688 cm^−1^, indicating the abundance of hydroxyl groups on the surfaces of T6 carbon dots. In the -OH envelope, several signals were observed to be more pronounced at 2921/2970 cm^−1^, which was attributed to C-H stretching vibrations in carboxylic acid chains. The latter’s contribution to the surface of T6 was confirmed by prominent peaks at 1737 and 1614 cm^−1^, associated with C=O and C-O stretching vibrations in the -COOH group. Therefore, the FTIR analysis of the T6 sample showed the presence of the polar functional group, which is responsible for the good water solubility of T6 carbon dots. Moreover, the diverse heterogeneity of the surface functionalization and the dominance of oxygen-related groups might contribute to the relatively low QY values observed in photoluminescent analysis [38,39,40].

## 4. Conclusions

In conclusion, this study demonstrates, for the first time, the potential of using humins derived from soybean peels as a precursor for carbon dots, comparing two distinct synthesis approaches: hydrothermal treatment and heat/sonication treatment.

The sonochemical method offers several advantages for synthesizing carbon dots, including rapid synthesis at mild temperatures, energy efficiency, and environmental friendliness. It enables precise control over particle size and optical properties, leading to carbon dots with high quantum yields. Additionally, this method is versatile, allowing the use of various precursors, including renewable biomass, and can be easily scaled up for large-scale production. In addition, our results was similar to those of a previous study [35] that demonstrated the formation of biocompatible nano-biomass dots from soybeans using a sonochemical approach.

The CDs with the best photochemical properties were those obtained with the sonochemical approach applied to humins derived from the dehydration of soybean peels with phosphoric acid and pretreatment. Such CDs, designated as the **T6** sample, showed a relative QY % around 2.5%. Carbon dots (CDs) possess unique fluorescence properties, high zeta potential, and the ability to be surface-functionalized, making them ideal for various biomedical applications. These include bioimaging, drug delivery, cancer therapy, and biosensing, with potential for theranostics in personalized treatments.

However, further studies are needed to enhance their fluorescence and assess their in vitro biocompatibility and cytotoxicity for potential applications in the biological field [41].

Additionally, CDs show promise in environmental applications, such as water quality monitoring, where their high and functionalizable surfaces enable the detection of pollutants like heavy metals, organic compounds, and microbes. Moreover, their excitation-dependent fluorescence and high zeta potential enhance both sensor sensitivity and interaction with positively charged pollutants.

Overall, the results obtained in this study demonstrate that humins can be effectively utilized for the production of versatile, high-value photoluminescent carbon dots through a straightforward methodology.

## Figures and Tables

**Table 1:** Summary of all the prepared materials.

**Humin Sample**	**Starting Material**	**Methodology**	**Pretreatment**	**Condition**	**Temp. and Time**
**H1**	Soybean peels		none	CH_3_COOH	200 °C 2 h
**H2**	Soybean peels		none	H_3_PO_4_	200 °C 2 h
**H3**	Soybean peels		H_3_PO_4_ (80 °C 2 h)	H_3_PO_4_	200 °C 2 h
**CD Sample**	**Starting Material**	**Methodology**	**Pretreatment**	**Condition**	**Temp. and Time**
**T1A**	H1	Hydrothermal		H_3_PO_4_	200 °C 3 h
**T1B**	H1	Hydrothermal		H_3_PO_4_	200 °C 6 h
**T2A**	H2	Hydrothermal		H_3_PO_4_	200 °C 3 h
**T2B**	H2	Hydrothermal		H_3_PO_4_	200 °C 6 h
**T3A**	H3	Hydrothermal		H_3_PO_4_	200 °C 3 h
**T3B**	H3	Hydrothermal		H_3_PO_4_	200 °C 6 h
**T4**	H1	Sonochemical	air 250 °C 2 h	550 W	RT 30′ min
**T5**	H2	Sonochemical	air 250 °C 2 h	550 W	RT 30′ min
**T6**	H3	Sonochemical	air 250 °C 2 h	550 W	RT 30′ min

**Table 2:** Description of the main signal of ATR-FTIR—spectra reported in Figure 5.

Signal (cm^−1^)	Description
3275	Stretching O-H
2854/2924	Symm. and asymm. CH_2_ stretching
1699	C=O stretching of conjugated aldehyde
750–800	C-H (furanic ring) out-of-plane bending

**Table 3:** Summary table of hydrothermal experiments.

Substrate	Catalyst	Sample Name	UV	Fluorescence	QY (%)
Soybean peel	Acetic acid	T1	Yes	Very low	//
Soybean peel	Phosphoric acid	T2	Yes	Very low	//
Soybean peel	Phosphoric acid and pretreatment	T3	Yes	Very low	//

**Table 4:** Summary table of sonochemical experiments.

Substrate	Catalyst	Sample Name	UV	Fluorescence	QY (%)
Soybean peel	Acetic acid	**T4**	Yes	Very low	//
Soybean peel	Phosphoric acid	**T5**	Yes	Very low	//
Soybean peel	Phosphoric acid and pretreatment	**T6**	Yes	Yes	2.6 ± 0.1 (As prepared) 2.4 ± 0.1 (After dialysis)

**Table 5:** Summary table of carbon dot synthesis processes found in the literature compared with the reaction carried out in this work.

Entry	Precursors	Method	Size (nm)	Quantum Yield (%)	Reference
1	Lettuce leaves	180 °C 6 h (hydrothermal)	2.8	0.38	[33]
2	Grape seeds	200 °C 8 h (hydrothermal)	8.9	0.275	[32]
3	Sugarcane bagasse	200 °C 12 h (hydrothermal)	From 2 to 8	0.091	[34]
4	Soybeans	Sonochemical 2 h	2.4	16.7	[35]
5	Soybean husks	Pretreatment (250 °C for 2 h) Microwave (450 W for 50 min)	Not reported ^a^	Not reported ^a^	[36]
6	Humins	(T1)200 °C 3 h (hydrothermal)	//	//	This work
7	Humins	Pretreatment (air 250 °C 2 h) Sonochemical method (550 W)	7.3 ± 1.2	2.6 ± 0.1	This work

^a^ visible fingerprint of 43.4%.

## Data Availability

The original contributions presented in this study are included in the article. Further inquiries can be directed to the corresponding author.
